# Mammary phyllodes tumor with six episodes of a relapse: a case report

**DOI:** 10.1186/s13256-017-1432-y

**Published:** 2017-09-15

**Authors:** Nozomi Iimori, Shinichiro Kashiwagi, Tetsuro Ishikawa, Hidemi Kawajiri, Tsutomu Takashima, Masahiko Ohsawa, Kosei Hirakawa, Masaichi Ohira

**Affiliations:** 1 Department of Surgical Oncology, Osaka City University Graduate School of Medicine, 1-4-3 Asahi-machi, Abeno-ku, Osaka 545-8585 Japan; 2 Department of Surgery, Kahiwara Municipal Hospital, 1-7-9 Houzenji, Kashiwara-shi, Osaka Japan; 3 Department of Breast and Neck Surgery, Ishikiri Seiki Hospital, 18-28 Yayoi-cho, Higashi Osaka-shi, Osaka Japan; 4 Department of Diagnostic Pathology, Osaka City University Graduate School of Medicine, 1-4-3 Asahi-machi, Abeno-ku, Osaka Japan

**Keywords:** Phyllodes tumor, Recurrence, Surgery, Breast tumor, Malignant

## Abstract

**Background:**

Phyllodes tumor is a rare breast mass. Most phyllodes tumors are benign, but occasionally some show malignancy. Even if the tumors are benign, they can easily show recurrence.

**Case presentation:**

We report a case of a 48-year-old Asian woman, who had previously undergone a tumorectomy of her left breast 12 years before, with a pathological diagnosis of fibroadenoma. Five years after the initial tumorectomy, the patient presented with an abnormally enlarged left breast. A biopsy determined the growth to be a phyllodes tumor; subsequently, a partial mastectomy was conducted. However, the patient’s left breast showed rapid enlargement in the next 5 months. The treating physicians suspected a relapse and subsequently consulted with our hospital. The breast mass was resected at our institution. After this surgery, the patient had repeated episodes of relapse and underwent four additional operations. Since then, the patient has not had any additional relapse so far.

**Conclusions:**

We present a case of a phyllodes tumor with multiple episodes of relapse. Although phyllodes tumors commonly show relapse, this case was unique because of the number of episodes of relapse. This case highlights the need to carry out tumorectomy with adequate margins with subsequent careful observation to check for relapse.

## Background

Phyllodes tumor is a rare tumor of the breast [1, 2]. Most cases are benign, but occasionally malignant phyllodes tumors are encountered [3, 4]. Identifying the malignant subtype is challenging because it shows a varying clinical course and histology. Some cases of this tumor with repeated episodes of relapse and a subsequent change from a benign to a malignant status have been reported [3, 4]. In this study, we report a case of a patient with phyllodes tumor with six episodes of relapse.

## Case presentation

A 48-year-old Asian woman initially underwent a tumorectomy 12 years ago, with a pathological diagnosis of fibroadenoma (pericanalicular pattern dominant). Five years later, the patient consulted another doctor regarding a growth in her left breast. A biopsy was performed, and a diagnosis of phyllodes tumor was made. The patient subsequently underwent a partial mastectomy. Five months after the mastectomy, her left breast began to show enlargement again. The attending physician suspected a relapse and proposed total mastectomy. The attending physician then referred the patient to our hospital for total mastectomy and reconstructive surgery.

**Fig. 1:**
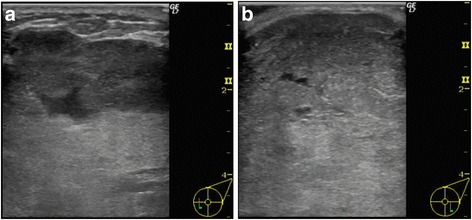
Ultrasonographic findings. **a**, **b** Ultrasonographic images showing a large segmental tumor in the left mammary gland

**Fig. 2:**
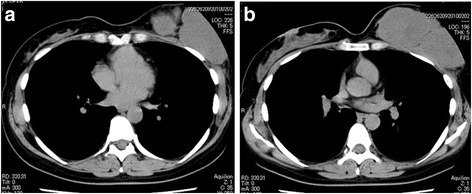
Computed tomographic findings. **a**, **b** Computed tomographic images showing a large tumor in the left breast. Distant metastases were not observed

**Fig. 3:**
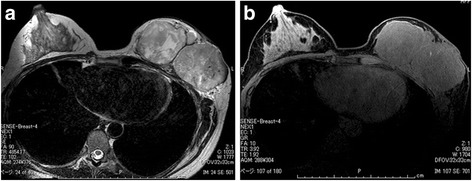
Magnetic resonance imaging findings. **a** The stroma shows high signal intensity on a T2-weighted image. **b** Magnetic resonance imaging scan showing a large tumor that was well-defined but attached to the greater pectoral muscle

**Fig. 4:**
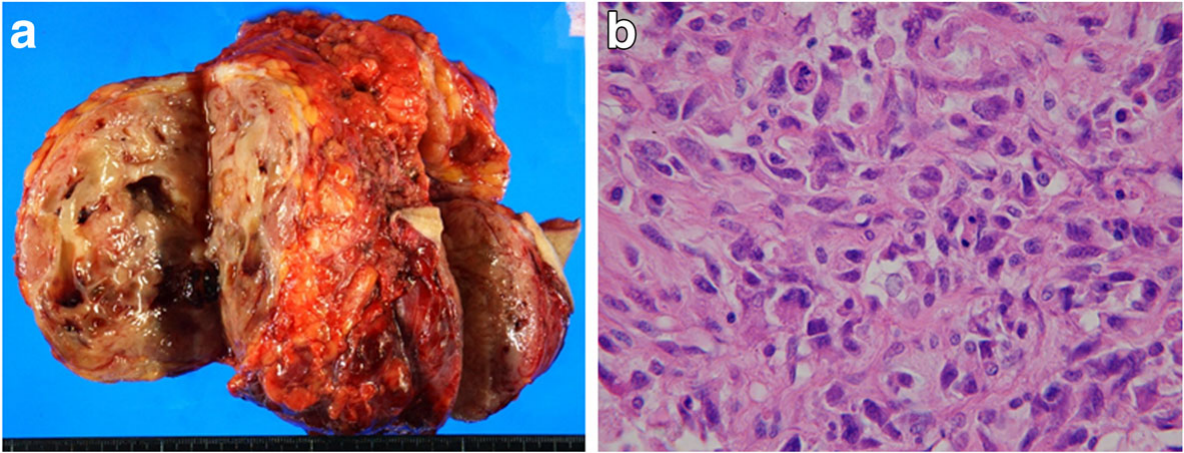
Macroscopic findings of the resected specimen. **a** The resected specimen measured 16×14 cm, and the cut surface was a lobulated solid tumor. **b** Histopathological findings. Mitoses were frequently observed in the interstitial cells, with recognition of polynuclear giant cells (hematoxylin and eosin stain, original magnification ×200)

The tumor of the left breast was elastic and hard, and it had mobility. The patient’s laboratory data were within normal ranges. Ultrasonographic images showed a large segmental tumor (Fig. 1a, b). The inside of the tumor was heterogeneous and had hypoechoic characteristics. Computed tomographic images also showed a large tumor of the left breast (Fig. 2a, b). There was no lymph node metastasis or distant metastasis. Magnetic resonance imaging scans showed a large tumor that was well-defined but attached to the greater pectoral muscle (Fig. 3a, b). We performed a total mastectomy and reconstructive surgery with the rectus abdominis muscle. The pathological diagnosis was malignant phyllodes tumor (Fig. 4a). There was a significant amount of mitosis but no evidence of infiltration to the surrounding muscle (Fig. 4b).

The patient had follow-up visits approximately 2–4 weeks after each surgery, with additional visits several months later. However, the patient often presented at our hospital prior to follow-up appointments with a chief complaint of breast enlargement. Because we could not predict relapse, it was not clear what follow-up period should be used. Ultimately, we decided to follow the patient within several weeks after the surgery. In total, the patient would have six episodes of relapse. The periods between consecutive episodes of relapse were short, with the smallest period being 20 days. The areas of relapse in the breast were different every time: sometimes under the nipple and another time near a drainage tube scar from a previous surgery. In every incident of relapse, the pathological diagnosis was always malignant phyllodes tumor. Despite attempting radiation therapy after previous surgeries, tumor control was not obtained. The sixth surgery was performed to treat a relapse under the nipple, with adjuvant radiation therapy also delivered. The patient currently lives relapse-free. Distant metastasis was not observed during any period in this case.

## Discussion

The proportion of phyllodes tumors among breast tumors is less than 1%, with an even lower frequency of malignant tumors [1, 2]. Regardless of whether they are benign or malignant, phyllodes tumors are prone to rapid growth [1, 3]. However, malignancy is doubtful when the tumor size is large and the growth rate is excessive. In this case, the tumor was determined to be malignant on the basis of pathological examination.

The pathologist also reported abnormal mitosis and a low nuclear grade [1, 5]. Phyllodes tumors consist of epithelial and stromal tissue, with some reports suggesting it is a type of fibroadenoma [5–7]. The first onset of this tumor was diagnosed as a fibroadenoma, which is difficult to distinguish from a phyllodes tumor [6, 8]. All pathological diagnoses in this case determined malignancy. Interestingly, the pathologists reported abnormal proliferation of stromal cells and no epidermal cells in the latter three episodes of relapse. It is possible that this tumor changed from a fibroadenoma to a malignancy with the change of proliferation in the stromal tissue and that malignancy was not initially detected.

To prevent a local relapse, surgeons must excise the tumor with sufficient margins [2, 9–12]. From this case, we learned that there is a possibility of relapse even from the scar of a drainage tube. This finding suggests that a very small number of tumor cells may cause relapse. It is advisable to thoroughly clean around and inside the wound and to maintain sterility during surgery. In addition, the role of adjuvant therapy (radiation therapy, chemotherapy, endocrine therapy) in treating phyllodes tumors remains unclear [13, 14]. Therefore, the probability of additional relapse is unknown, and regular follow-up is necessary for adequate surveillance.

## Conclusions

We present a case of phyllodes tumor with six incidences of relapse. Phyllodes tumors can easily relapse, but the large number of episodes of relapse that occurred in this case is rare. This case highlights the need to carry out tumorectomy with adequate margins with subsequent careful observation to check for relapse.
